# Association between helminth infection and impaired SARS-CoV-2 antibody responses following COVID-19 vaccination: a cross-sectional study in Malawi

**DOI:** 10.1038/s41598-026-62359-9

**Published:** 2026-07-24

**Authors:** Mhairi J. McCormack, Louis Banda, Stephen Kasenda, Lyson Samikwa, Harry Meleke, Ellen C. Hughes, Amelia Crampin, David Chaima, Tonney Nyirenda, Brian J. Willett, Abena S. Amoah, Antonia Ho

**Affiliations:** 1 MRC-University of Glasgow Centre for Virus Research, Glasgow, G61 1QH UK; 2 Malawi Epidemiology and Intervention Research Unit (MEIRU), Karonga, Malawi; 3 Kamuzu University of Health Sciences (KUHeS), Blantyre, Malawi; 4 The Roslin Institute and R(D)SVS, University of Edinburgh, Edinburgh, UK; 5 School of Health and Wellbeing, University of Glasgow, Glasgow, UK; 6 Department of Population Health, Faculty of Epidemiology and Population Health, London School of Hygiene and Tropical Medicine, London, UK; 7 Leiden University Center for Infectious Diseases, Leiden University Medical Center, Leiden, The Netherlands

**Keywords:** SARS-CoV-2, Helminth, Neutralisation, Antibody, Vaccination, Diseases, Immunology, Medical research, Microbiology

## Abstract

Chronic helminth infections can modulate immune responses to infection and vaccination. This study explored the association between current helminth infection, including waterborne and soil-transmitted helminths (STHs), and severe acute respiratory syndrome coronavirus 2 (SARS-CoV-2) neutralising and IgG antibody responses. Samples were collected cross-sectionally from participants across rural (Karonga) and urban (Lilongwe) regions in Malawi. Helminth infections were detected via real-time PCR in stool and urine samples, targeting *Schistosoma* spp., *Ascaris lumbricoides*, *Ancylostoma duodenale*, *Necator americanus*, and *Trichuris trichiura*. SARS-CoV-2 nAbs were measured using a human immunodeficiency virus (HIV)-based pseudotyped virus neutralisation assay. A nucleocapsid (N) protein enzyme-linked immunosorbent assay (ELISA) identified prior natural SARS-CoV-2 infection in vaccinated participants. IgG targeting the spike (S) protein was measured using an ELISA. Helminth infection was found to be associated with reduced nAb responses post coronavirus disease 2019 (COVID-19) vaccination, but not after natural SARS-CoV-2 infection. Generalised additive model analysis confirmed this interaction while considering covariates. IgG responses were also impaired among those helminth-infected. It should be investigated whether integrating pre-vaccination deworming improves vaccine responses in helminth-endemic regions.

**Supplementary Information:**

The online version contains supplementary material available at 10.1038/s41598-026-62359-9.

## Introduction

Chronic helminth infections are known to modulate host immune responses to vaccines and pathogens, including SARS-CoV-2^1^. Helminth-induced immunoregulation may reduce COVID-19 severity by dampening hyperinflammatory responses^2^, yet the same regulation may also weaken vaccine-induced immunity^1^. This is particularly relevant in sub-Saharan Africa, where helminth infections are endemic and may partly contribute to the comparatively lower burden of severe COVID-19 in the region. Mbow et al.^3^ proposed that helminths promote IL-4–driven expansion of virtual memory T cells and reduce proinflammatory monocytes, thereby dampening cytokine storms and lung inflammation. Understanding these interactions is important for optimising COVID-19 control measures and vaccination strategies in helminth-endemic regions.

However, evidence on helminth–SARS-CoV-2 interactions remains limited. Two studies in Ethiopia reported reduced COVID-19 severity among helminth-infected individuals^2,4^, though neither examined SARS-CoV-2 antibodies. Other studies found no effect of infections with *Ascaris lumbricoides*, *Opisthorchis viverrine, Onchocerca volvulus,* or *Trichinella spiralis* on SARS-CoV-2 neutralising antibody (nAb) levels^5–8^, although methodologies varied. The *Ascaris*, *Onchocerca, and Trichinella* studies used serology to infer helminth exposure rather than demonstrating active infection^5,7,8^, while the Opisthorchis study measured active infection and reported reduced receptor binding domain (RBD)-specific IgG levels in co-infected individuals^6^. One study observed impaired anti-SARS-CoV-2 spike protein S1 subunit IgG responses in individuals with moderate or severe *Schistosoma mansoni* infection compared with those Kato-Katz negative^9^, however they had very limited participants, and did not assess functional immunity. Schistosome-derived molecules, such as omega-1^10^ and IPSE/alpha-1^11^, are known to have immunomodulatory properties that are likely involved in this reduced antibody response in *S. mansoni* infected individuals. Additionally, a recent study in Ghana reported higher helminth seroprevalence among COVID-19 asymptomatic individuals and an associated shift toward Th2-type immune responses^12^.

Here, we investigated whether current helminth infections, as determined by real time PCR, were associated with altered SARS-CoV-2 neutralising or IgG antibody responses in adults from rural and urban Malawi, hypothesising that *Schistosoma* spp. is the primary driver of this immunomodulation. These data provide insights into SARS-CoV-2 immunity in helminth-endemic settings and have implications for vaccination programmes.

## Methods

### Study population

The study was conducted in Karonga (rural) and Lilongwe (urban, Area 25) (Supplementary Fig. 1), where *Schistosoma* spp. and soil-transmitted helminths (STHs) are endemic^13–15^. We included 371 adults participating in a longitudinal SARS-CoV-2 serosurveillance cohort^16^. Samples were collected cross-sectionally between January and April 2022 with written, informed consent. Individuals with HIV were excluded due to known interference of antiretroviral therapy with the pseudotyped virus neutralisation assay^17^. Sample size was constrained by funding, and power calculations indicated that the study was only powered to detect moderate to large differences in SARS-CoV-2 antibody titres (Supplementary Methods).

### Sample collection and laboratory procedures

Questionnaires captured demographic and clinical data (Supplementary Methods). Blood, stool and urine samples were collected. Serum was stored at –80 °C before transfer to the MRC-University of Glasgow Centre for Virus Research for SARS-CoV-2 assays. Stool and urine samples were stored at –80 °C and analysed at Kamuzu University of Health Sciences for helminth infection.

Neutralisation against ancestral B.1, Beta, Delta, Omicron BA.1, and BA.2 variants (Supplementary Table 1), the predominant variants circulating in Malawi before sample collection^16^, was measured using an HIV pseudotype-based assay. All participants were also tested for anti-spike (S) IgG by ELISA to measure the overall IgG responses (Supplementary Methods). Vaccinated participants were tested for anti-nucleocapsid (N) IgG by ELISA (Supplementary Methods) to distinguish who had also been naturally SARS-CoV-2 infected in addition to their vaccination-induced immune responses. Because both vaccines administered in this cohort (Janssen-Cilag International NV Ad26.COV2-S vaccine (J&J) and AstraZeneca-Oxford vaccine AZD1222 Vaxzeria (AZ)) do not include the N protein, N-antibody negativity in vaccinated individuals was taken to indicate vaccination-only status. In contrast, N-antibody positivity was interpreted as evidence of prior natural infection in addition to vaccination (hybrid immunity). Participants with no reported vaccination were classified as infection-only. These serological definitions were used to stratify analyses of antibody responses, enabling comparison between SARS-CoV-2 infection-only, vaccination-only, and hybrid immunity groups.

DNA extracted from stool and urine was tested by real-time PCR for *Schistosoma* spp., *A. lumbricoides, Ancylostoma duodenale, Necator americanus*, and *Trichuris trichiura*. Urine samples were tested for S*chistosoma* spp. (Supplementary Methods, Supplementary Table 2).

### Statistical analyses summary

For prevalence percentages, 95% binomial confidence intervals (CI) were reported. A two-proportion chi-squared test (two-tailed) was used to compare prevalence between groups. For nAb titres, the median and interquartile range (IQR) were presented as the data were not normally distributed. The non-parametric two-tailed Wilcoxon rank-sum test was used to assess statistical differences in titres. A false discovery rate (Benjamini-Hochberg) correction was applied when making multiple individual comparisons to reduce false positivity, while maintaining statistical power for the small sample size. P-values < 0.05 were considered statistically significant, with very small p-values represented as p < 0.0001. Further statistical analysis methods that involved descriptive analysis of participants and effect size estimate methods are described in the Supplementary Methods. Generalised additive models (GAMs) were used to evaluate predictors of nAb titres, including age, helminth infection status, vaccination, and time since vaccination (additional model methodological details in Supplementary Methods). A GAM was chosen to assess the influence of covariates, as initial linear regression analysis displayed poor predictive power and indicated the presence of non-linear relationships. Within the model, the F-test was used to evaluate the significance and fit of continuous predictor variables, whereas the Wald test was used to test the significance of categorical predictor variables.

## Results

### Participant characteristics

Of 371 participants, 242 were from Karonga (rural) and 129 were from Lilongwe (urban). Median age was similar between sites (Karonga: 36.1 years, IQR 16.0–50.0 years; Lilongwe: 38.2 years, IQR 28.3–49.2 years; p = 0.49, Wilcoxon test) (Supplementary Table 3). A lower proportion of participants in Karonga were female (p = 0.002, χ^2^ test). COVID-19 vaccination coverage was 31.8% (95% CI 26.3–37.9) in Karonga and 34.9% (95% CI 27.2–43.4) in Lilongwe. Among those vaccinated, 28.7% had received one dose of AZ vaccine, 50% had received two doses of the AZ vaccine, and 21.3% had received one dose of the J&J vaccine, all derived from the ancestral B.1 strain. Self-reported praziquantel treatment for schistosomiasis was more frequently reported in Karonga, whereas reported symptoms potentially related to helminth infection (blood in stool or urine, and pain on urination) were comparable between sites (Supplementary Table 3).

### Helminth infection prevalence and species distribution

**Fig. 1.**
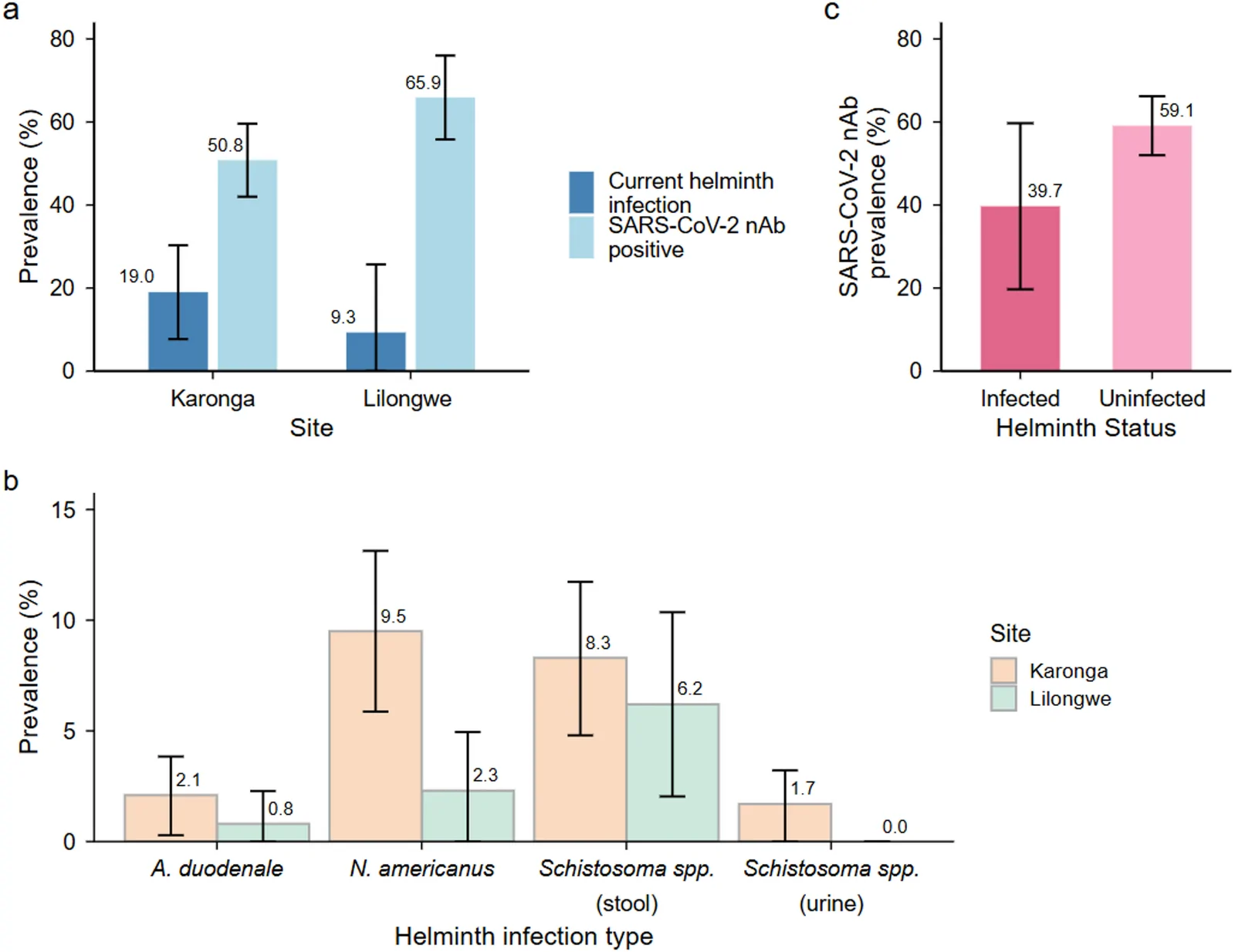
Prevalence of helminth infections and SARS-CoV-2 neutralising antibodies (nAbs) in the cohort. (**a**) Prevalence by site (Karonga–rural; Lilongwe–urban) of helminth infections (dark blue) and SARS-CoV-2 nAbs (light blue). (**b**) Prevalence of each type of helminth per site (Karonga–orange; Lilongwe–green). (**c**) SARS-CoV-2 nAb prevalence by helminth status (infected–dark pink; uninfected–light pink). Bars show percentages with 95% CI error bars.

Overall helminth prevalence (PCR-positive for ≥ 1 species) was higher in Karonga (19.0%, 95% CI 7.7–30.3) compared with Lilongwe (9.3%, 95% CI 0.0–25.7) (p = 0.021, χ^2^ test) (Fig. 1a). The most frequently detected species were the hookworm *N. americanus* (hookworm) and *Schistosoma* spp. (Fig. 1b). Neither *A. lumbricoides* nor *T. trichiura* were detected.

Six individuals had multiple helminth infections: two were PCR-positive for *Schistosoma* spp. in both stool and urine, three were positive for *N. americanus* and *Schistosoma* spp. in stool, and one was positive for both common species of hookworm, *N. americanus* and *A. duodenale*. Helminth prevalence did not differ by age, sex, or reported water contact (Supplementary Table 4, Supplementary Figs. 2,3).

### SARS-CoV-2 nAb prevalence by site and helminth status

SARS-CoV-2 nAbs were detected more frequently in Lilongwe (65.9%, 95% CI 55.8–76.0) than in Karonga (50.8%, 95% CI 42.0–59.6) (Fig. 1a). Participants without helminth infection had a higher nAb prevalence (59.1%, 95% CI 52.0–66.2) compared with helminth-infected participants (39.7%, 95% CI 19.7–59.7; p = 0.0094, χ^2^ test) (Fig. 1c).

### Helminth infection impairs SARS-CoV-2 nAb response

Overall, nAb titres were significantly higher in helminth-uninfected individuals (median = 314, IQR = 119–907) than in those infected with helminths (median = 129, IQR = 60.7–405, p < 0.0001, Wilcoxon rank-sum test). Although the difference was statistically significant, the effect size was small (Cliff’s δ = 0.295). A bootstrap simulation indicated 99.8% power to detect this difference.

### Vaccinated individuals exhibited greater helminth-associated reduction in SARS-CoV-2 nAbs

**Fig. 2.**
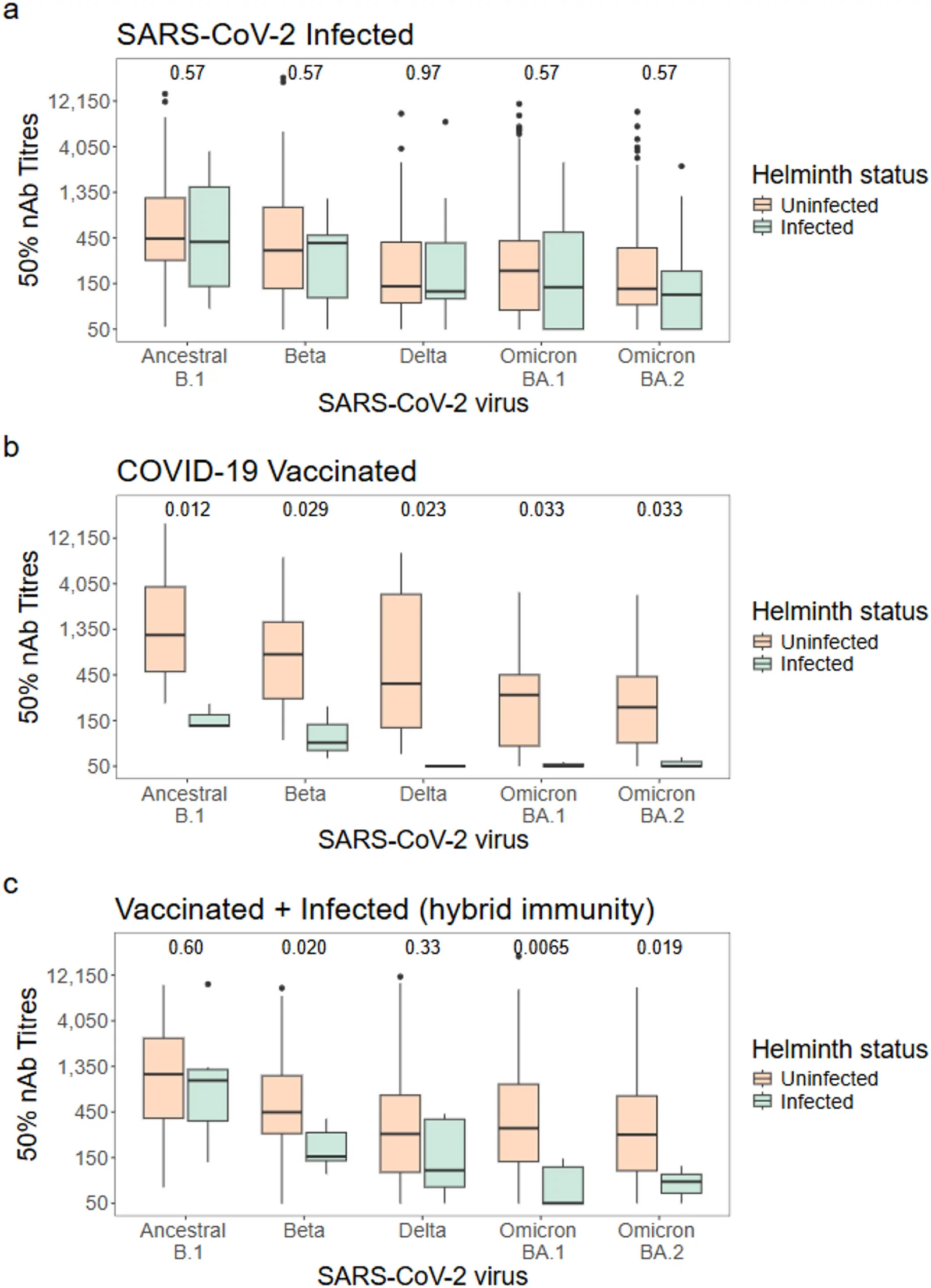
SARS-CoV-2 neutralising antibody (nAb) titres by helminth status across SARS-CoV-2 exposure histories. (**a**) SARS-CoV-2 infected only (n = 109) — n = 96 helminth-uninfected; n = 13 helminth-infected. (**b**) COVID-19 vaccinated only (n = 18) — n = 15 helminth-uninfected; n = 3 helminth-infected. (**c**) Vaccinated and infected (hybrid immunity; n = 76) — n = 69 helminth-uninfected; n = 7 helminth-infected. Antibody titres against each of the SARS-CoV-2 variants were measured using an HIV(SARS-CoV-2) pseudotyped virus neutralisation assay. Boxplots show median and IQR of 50% titres. The whiskers stretch to the farthest data points that lie within 1.5 × IQR of Q1 and Q3, while any points beyond this range are displayed as outliers. Wilcoxon (rank-sum) test with false discovery rate correction was used; p-values shown. Due to very small subgroup sizes, statistical comparisons should be interpreted with caution and are presented as exploratory analyses.

Participants were grouped by SARS-CoV-2 exposure history and variant: for those with natural infection (n = 109), nAb titres did not differ by helminth status for any variant assessed (p > 0.05 – Wilcoxon rank-sum tests) (Fig. 2a). For COVID-19 vaccinated individuals (n = 18), those without helminth infection had substantially higher titres across all variants (Fig. 2b). For the ancestral B.1 strain, the median nAb titre among uninfected participants was 1177.2 (IQR 498.0–3887.1), compared with 132.8 (IQR 130.9–178.7) in helminth-infected individuals, a statistically significant difference (p = 0.012, Wilcoxon rank-sum test). Similar patterns were observed for other variants: Beta (739.8, IQR 255.4–1718.1 vs 88.4, IQR 74.5–149.8; p = 0.029), Delta (365.5, IQR 126.7–3169.7 vs 50, IQR 50–50; p = 0.023), Omicron BA.1 (278.4, IQR 81.6–451.7 vs 50, IQR 50–52.6; p = 0.033), and Omicron BA.2 (206.5, IQR 92.6–476.9 vs 50, IQR 50–56.1; p = 0.033), all assessed using Wilcoxon rank-sum tests. Among individuals with hybrid immunity (both SARS-CoV-2 infected and COVID-19 vaccinated; n = 76), helminth-uninfected participants had significantly higher nAb titres than helminth-infected individuals across multiple variants (Beta: 447.0, IQR 267.8–1078.8 vs 154.2, IQR 139.1–278.2, p = 0.020; Omicron BA.1: 304.5, IQR 136.2–877.8 vs 50, IQR 50–119.8, p = 0.0065; Omicron BA.2: 261.2, IQR 109.2–662.6 vs 84.0, IQR 63.8–100.9, p = 0.019) (Wilcoxon rank-sum tests; Fig. 2c).

### Helminth species effect

Contrary to our initial hypothesis that *Schistosoma* spp. was the primary driver of immunomodulation, SARS-CoV-2 nAb titres were not found to differ between participants infected with STHs (*A. duodenale* and *N. americanus*; n = 13) and those infected with *Schistosoma* spp. (n = 9) (p > 0.6 for all variants—Wilcoxon rank-sum test; Supplementary Fig. 4), though sample sizes were small.

### Multivariable analysis of SARS-CoV-2 nAb determinants

In the multivariate GAM including all individuals with detectable nAbs (n = 203), age and the interaction between helminth status and COVID-19 vaccination were significant predictors of SARS-CoV-2 nAb responses. Vaccinated, helminth-infected individuals had 41% (95% CI 22–55%, p = 0.00017, Wald test) lower nAb titres than vaccinated, helminth-uninfected individuals (Supplementary Table 5). There was a significant linear relationship between age and nAb titres among these individuals with SARS-CoV-2 responses (n = 203) (estimated degrees of freedom (EDF) = 1, χ^2^ = 30.3, p < 0.0001, F-test) indicating that as age increased, nAb levels also increased (Supplementary Table 6, Supplementary Fig. 5).

A GAM model restricted to vaccinated participants (n = 94) identified helminth status and time since COVID-19 vaccination as significant predictors of SARS-CoV-2 nAb responses. Helminth-infected individuals had 29% lower titres (95% CI 16–41%, p = 0.0018, Wald test) (Supplementary Table 7). There was also a significant non-linear relationship between time since vaccination and nAb titres among vaccinated individuals (n = 94) (EDF = 6.72, χ^2^ = 46.8, p < 0.0001, F-test) (Supplementary Table 8). Neutralisation titres rose until ~ 100 days post-vaccination, remained stable until ~ 300 days, then began to decline (Supplementary Fig. 6).

### SARS-CoV-2 IgG responses by helminth infection status

**Fig. 3.**
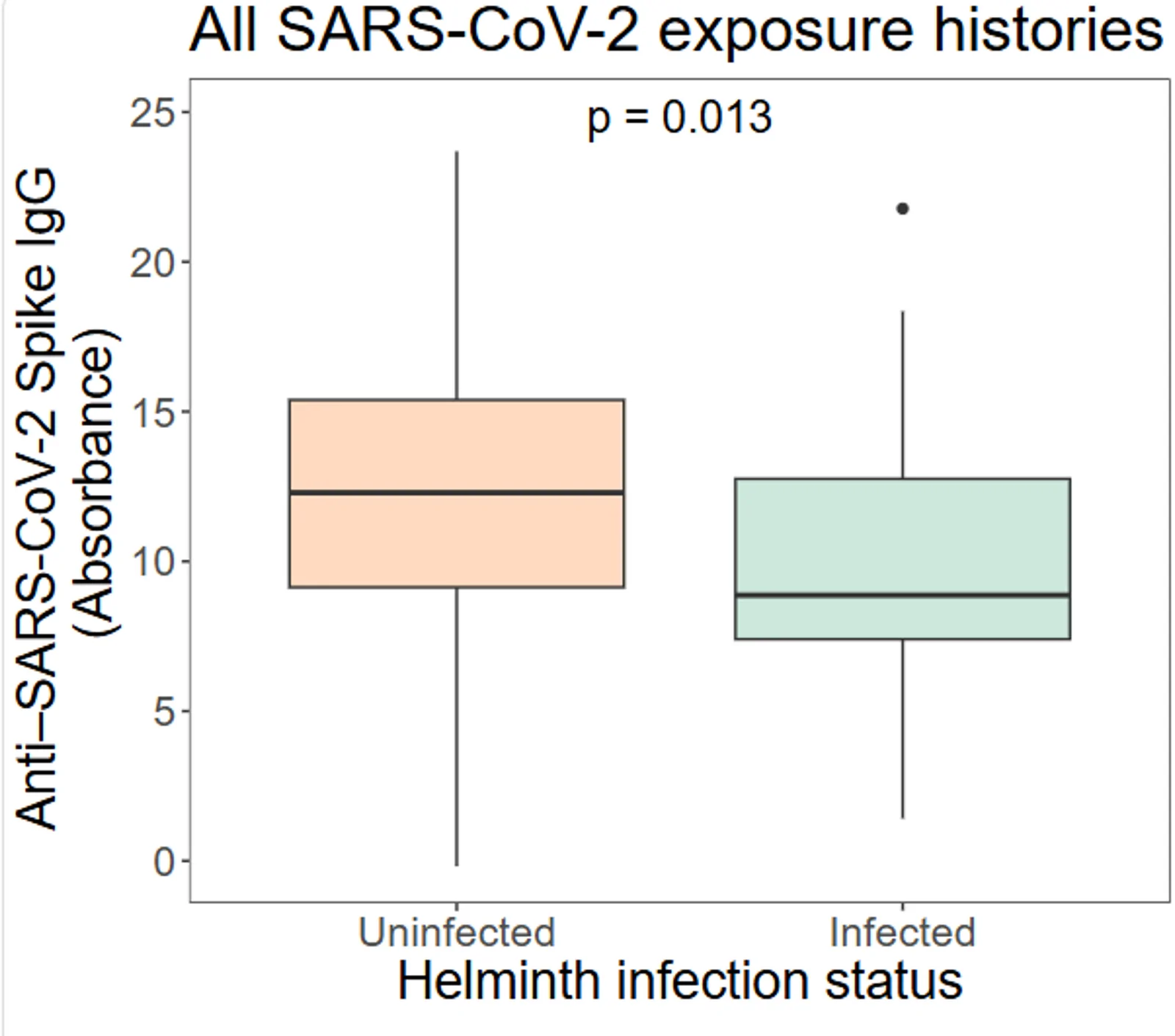
Anti-SARS-CoV-2 Spike (S) IgG (Absorbance) by helminth status among those S ELISA positive. Anti-SARS-CoV-2 S IgG measured with an enzyme-linked immunosorbent assay (ELISA) using the ancestral B.1 S protein. 204 participants were helminth-uninfected, 34 were helminth-infected. Boxplots show median and IQR of 50% titres. The whiskers stretch to the farthest data points that lie within 1.5 × IQR of Q1 and Q3, while any points beyond this range are displayed as outliers. Wilcoxon (rank-sum) test was used; p-values shown.

Among all SARS-CoV-2 exposed individuals (n = 238), helminth-infected individuals (n = 34) had lower anti-S IgG levels (median 8.87, IQR 7.40–12.8) than helminth-uninfected individuals (n = 204) (median 12.3, IQR 9.13–15.4; p = 0.013 – Wilcoxon rank-sum test) (Fig. 3). However, when stratified by SARS-CoV-2 exposure history, differences in IgG responses by helminth status were not statistically significant (p > 0.05 for all comparisons – Wilcoxon rank-sum test) (Supplementary Fig. 7). For those COVID-19 vaccinated, helminth-infected individuals had a median absorbance of 7.88 (IQR 6.76 to 10.3), while those helminth-uninfected had a median absorbance of 13.9 (IQR 6.76 to 10.3) (p = 0.41 – Wilcoxon rank-sum test).

## Discussion

In this cross-sectional study of adults from rural and urban Malawi, helminth infection was associated with reduced SARS-CoV-2 neutralising and IgG antibody responses. The impairment in nAb responses among helminth-infected individuals was particularly evident following COVID-19 vaccination, but not after natural SARS-CoV-2 infection. This pattern is consistent with existing literature demonstrating that chronic helminth infections can impair vaccine-induced immunity through immunoregulatory pathways, including IL-10 production and modified Th2 responses^18,19^.

Helminth species distribution in our cohort was broadly comparable to national trends, although some differences were observed. *N. americanus* prevalence was lower in our study than in the Malawian DeWorm3 cohort^20^, whereas *A. duodenale* was more common. *T. trichiura* and *A. lumbricoides* were rare, with no cases detected in this cohort. Notably, *S. mansoni* prevalence was higher than *S. haematobium* in our cohort, despite the latter being more prevalent nationally^21^. Although *Schistosoma* spp. prevalence and symptom reporting were similar across sites, access to praziquantel treatment was lower in urban Lilongwe. This likely reflects historical prioritisation of mass drug administration programmes towards rural and lakeside populations considered high risk, potentially overlooking persistent transmission in urban and non–lake-residing populations^22^.

Our findings differ from previous studies that reported no effect of helminth infections on SARS-CoV-2 nAb responses, however the studies assessed helminth species individually, specifically *A. lumbricoides*^5^, *O. viverrini*^6^ and *O. volvulus*^7^, and *T. spiralis*^8^ infections. We did not test for *O. viverrine, O. volvulus,* or *T. spiralis* in our study. Several factors may explain the discrepancies with the *A. lumbricoides* study. Firstly, the prevalence of *A. lumbricoides* among our participants was low, with *Schistosoma* spp. and *N. americanus* dominating the helminth infections. Second, the *A. lumbricoides* study only examined naturally infected participants^5^, whereas our strongest effects were observed after vaccination. Third, differences in diagnostic approaches, serology vs. PCR, may influence classification of active vs. past infection. Finally, immune interactions may vary between helminth species and across epidemiological settings. Importantly, although we found no species-specific differences in neutralisation between STHs and *Schistosoma* spp*.*, the small sample size limited our ability to draw firm conclusions. We initially hypothesised that the decreased nAb titres to SARS-CoV-2 were driven by the immunomodulatory properties of schistosome-derived molecules, such as omega-1^10^ and IPSE/alpha-1^11^. However, multiple helminth species are known to induce overlapping regulatory pathways^23^; therefore, shared immunomodulatory effects, rather than species-specific mechanism may underlie the observed impairment of vaccine induced immunity. Consistent with this, chronic helminth infections in humans are associated with a broader immune-regulatory or “modified Th2” phenotype, characterised increased IL-10 production and IgG4 responses rather than a purely IL-4–driven response^18^, which could suppress SARS-CoV-2 nAb generation.

Our multivariable analyses identified the interaction between helminth infection and COVID-19 vaccination, but not natural infection, as a key determinant of neutralisation. This supports evidence from animal and human studies showing attenuated vaccine responses in the presence of helminth infection^1,19,24^. All participants received adenoviral-vector vaccines, which typically elicit lower neutralisation antibody levels than mRNA vaccines^25^. It is possible that helminth-related suppression may be less pronounced with mRNA platforms. Vaccination coverage in our cohort was modest (31.8% in Karonga; 34.9% in Lilongwe), consistent with national challenges during the study period, including limited vaccine supply and hesitancy. Such coverage may have limited population-level herd immunity, increasing reliance on infection-derived immunity and contributing to the observed neutralisation patterns.

Spike IgG responses mirrored the neutralisation results, with overall lower SARS-CoV-2 IgG levels among helminth-infected individuals, although differences were not significant when stratified by exposure. Small sample size likely contributed, but trends suggest that helminth infection modulates SARS-CoV-2 immunity in a context-dependent manner, with the clearest effects following vaccination.

Strengths of this study include the use of functional neutralisation assays across multiple variants, inclusion of both rural and urban populations, and adjustment for key confounders, such as age and time since COVID-19 vaccination. Limitations include the cross-sectional design (which precludes assessment of dynamic immune responses), exclusion of children and people living with HIV, lack of nutritional data (such as body mass index (BMI)), and insufficient power to compare vaccine platforms or dose. PCR detection captured active helminth infections, but not cumulative exposure, which may be immunologically relevant. Lastly, the study had limited power to assess species-specific effects and did not include data on immune mediators, such as cytokines or chemokines, limiting mechanistic insight.

Overall, our findings suggest that helminth infection may impair COVID-19 vaccine responses in helminth-endemic regions. This has implications not only for COVID-19, but potentially for other vaccines deployed in similar settings^26,27^. Larger, longitudinal studies are needed to determine whether pre-vaccination deworming would improve vaccine immunogenicity, as prior work shows deworming can partially reverse immune inhibition^1^ and enhance oral cholera vaccine effectiveness^28^. Evaluating alternative vaccine platforms, including mRNA vaccines, may also be informative.

In summary, helminth infection was associated with reduced SARS-CoV-2 antibody responses, particularly reduced neutralisation following COVID-19 vaccination. Future work should assess the potential benefits of integrating pre-vaccination deworming with vaccination programmes and identify vaccine strategies that provide optimal protection in helminth-infected populations.

## Supplementary Information

Supplementary Information.

## Data Availability

Requests for individual, pseudo-anonymised data and supporting documents can be sent to [info@meiru.mw] , referencing the paper title. A data transfer agreement will be required.

## References

[1] Zhu, F. et al. *Trichinella spiralis* infection inhibits the efficacy of RBD protein of SARS-CoV-2 vaccination via regulating humoral and cellular immunity. *Vaccines (Basel)*10.3390/vaccines12070729 (2024). 39066367 10.3390/vaccines12070729 PMC11281533

[2] Wolday, D. et al. Effect of co-infection with intestinal parasites on COVID-19 severity: A prospective observational cohort study. *EClinicalMedicine* **39**, 101054. 10.1016/j.eclinm.2021.101054 (2021). 34368662 10.1016/j.eclinm.2021.101054 PMC8324426

[3] Mbow, M. et al. COVID-19 in Africa: Dampening the storm?. *Science* **369**, 624–626. 10.1126/science.abd3902 (2020). 32764055 10.1126/science.abd3902

[4] Toru, M., Atnaf, A., Mengist, H. M. & Reta, A. The COVID-19 severity and its association with intestinal parasite coinfection and urine biochemical parameters among COVID-19-confirmed patients admitted to Debre Markos University COVID-19 Center, Northwest Ethiopia. *Biomed. Res. Int.* **2024**, 3064374. 10.1155/2024/3064374 (2024). 38249633 10.1155/2024/3064374 PMC10799708

[5] Adjobimey, T. et al. Negative association between *Ascaris lumbricoides* seropositivity and Covid-19 severity: Insights from a study in Benin. *Front. Immunol.* **14**, 1233082. 10.3389/fimmu.2023.1233082 (2023). 37622109 10.3389/fimmu.2023.1233082 PMC10446766

[6] Charoensuk, L., Pinlaor, S., Nimala, B., Suttiprapa, S. & Prakobwong, S. Characteristics of SARS-CoV-2 and *Opisthorchis viverrini* coinfections: Insights into immune responses and clinical outcomes. *Parasitol. Res.* **123**, 297. 10.1007/s00436-024-08317-8 (2024). 39120805 10.1007/s00436-024-08317-8

[7] Meyer, J. et al. Humoral immune response to COVID-19 vaccines in onchocerciasis-infected individuals: a field study from Ghana. *Front. Immunol.* **16**, 1633187. 10.3389/fimmu.2025.1633187 (2025). 41103440 10.3389/fimmu.2025.1633187 PMC12521167

[8] Mitic, I. et al. Patients with *Trichinella spiralis* infection display unmodified antigen-specific immune response to SARS-CoV-2. *Mem. Inst. Oswaldo Cruz.* **120**, e250044. 10.1590/0074-02760250044 (2025). 41124407 10.1590/0074-02760250044 PMC12543365

[9] Niu, M. et al. COVID-19 vaccination survey and anti-SARS-CoV-2 IgG responses in a human cohort from *Schistosoma mansoni*-endemic villages in Mayuge District, Uganda: A cross-sectional study. *Front. Public Health* **12**, 1437063. 10.3389/fpubh.2024.1437063 (2024). 39624416 10.3389/fpubh.2024.1437063 PMC11609215

[10] Ferguson, B. J. et al. The *Schistosoma mansoni* T2 ribonuclease omega-1 modulates inflammasome-dependent IL-1β secretion in macrophages. *Int. J. Parasitol.* **45**, 809–813. 10.1016/j.ijpara.2015.08.005 (2015). 26385440 10.1016/j.ijpara.2015.08.005

[11] Knuhr, K. et al. *Schistosoma mansoni* egg-released IPSE/alpha-1 dampens inflammatory cytokine responses via basophil interleukin (IL)-4 and IL-13. *Front. Immunol.* **9**, 2293. 10.3389/fimmu.2018.02293 (2018). 30364177 10.3389/fimmu.2018.02293 PMC6191518

[12] Fogang, B. A. N. et al. Helminth seropositivity inversely correlated with Th1 and Th17 cytokines and severe COVID-19. *Vaccines (Basel)*10.3390/vaccines13030252 (2025). 40266113 10.3390/vaccines13030252 PMC11946601

[13] Kayuni, S. A. et al. An outbreak of intestinal schistosomiasis, alongside increasing urogenital schistosomiasis prevalence, in primary school children on the shoreline of Lake Malawi, Mangochi District. *Malawi. Infect. Dis. Poverty.* **9**, 121. 10.1186/s40249-020-00736-w (2020). 32867849 10.1186/s40249-020-00736-w PMC7456765

[14] The DeWorm3 Trials Team. Baseline patterns of infection in regions of Benin, Malawi and India seeking to interrupt transmission of soil transmitted helminths (STH) in the DeWorm3 trial. *PLoS Negl. Trop. Dis.* **14**, e0008771. 10.1371/journal.pntd.0008771 (2020). 33137100 10.1371/journal.pntd.0008771 PMC7673551

[15] Nyangulu, W. et al. The prevalence of *Schistosoma mansoni* infection among adults with chronic non-communicable diseases in Malawi. *Trop. Med. Health* **50**, 56. 10.1186/s41182-022-00450-3 (2022). 35986382 10.1186/s41182-022-00450-3 PMC9389769

[16] Banda, L. et al. Characterizing the evolving SARS-CoV-2 seroprevalence in urban and rural Malawi between February 2021 and April 2022: A population-based cohort study. *Int. J. Inf. Dis* **137**, 118–125. 10.1016/j.ijid.2023.10.020 (2023). 38465577 10.1016/j.ijid.2023.10.020 PMC10695832

[17] McCormack, M. J. et al. Retrovirus-based pseudotyped virus neutralisation assays overestimate neutralising activity in sera from participants receiving integrase inhibitors. *Sci. Rep.* **5**, 28580. 10.1038/s41598-025-11362-7 (2025). 10.1038/s41598-025-11362-7 PMC12325791 40764781

[18] Figueiredo, C. A. et al. Chronic intestinal helminth infections are associated with immune hyporesponsiveness and induction of a regulatory network. *Infect. Immun.* **78**, 3160–3167. 10.1128/iai.01228-09 (2010). 20404082 10.1128/IAI.01228-09 PMC2897394

[19] Desai, P. et al. Intestinal helminth infection impairs vaccine-induced T cell responses and protection against SARS-CoV-2 in mice. *Sci. Transl. Med.* **16**, eado1941. 10.1126/scitranslmed.ado1941 (2024). 39167662 10.1126/scitranslmed.ado1941

[20] Ajjampur, S. S. R. et al. Feasibility of interrupting the transmission of soil-transmitted helminths: The DeWorm3 community cluster-randomised controlled trial in Benin, India, and Malawi. *Lancet* **406**, 475–488. 10.1016/S0140-6736(25)00766-4 (2025). 40752908 10.1016/S0140-6736(25)00766-4

[21] Bowie, C., Purcell, B., Shaba, B., Makaula, P. & Perez, M. A national survey of the prevalence of schistosomiasis and soil transmitted helminths in Malawi. *BMC Infect. Dis.* **4**, 49. 10.1186/1471-2334-4-49 (2004). 15546483 10.1186/1471-2334-4-49 PMC535894

[22] Ministry of Health Malawi. Malawi Neglected Tropical Diseases Master Plan 2023–2030, https://espen.afro.who.int/sites/default/files/content/document/Malawi%20NTD%20Master%20Plan%202023-2030.pdf (2024).

[23] Maizels, R. M., Smits, H. H. & McSorley, H. J. Modulation of host immunity by helminths: the expanding repertoire of parasite effector molecules. *Immunity* **49**, 801–818. 10.1016/j.immuni.2018.10.016 (2018). 30462997 10.1016/j.immuni.2018.10.016 PMC6269126

[24] Hartmann, W. et al. Helminth infections suppress the efficacy of vaccination against seasonal influenza. *Cell Rep.* **29**, 2243-2256.e2244. 10.1016/j.celrep.2019.10.051 (2019). 31747598 10.1016/j.celrep.2019.10.051

[25] Davis, C. et al. Reduced neutralisation of the Delta (B.1.617.2) SARS-CoV-2 variant of concern following vaccination. *PLoS Pathog.* **17**, e1010022. 10.1371/journal.ppat.1010022 (2021). 34855916 10.1371/journal.ppat.1010022 PMC8639073

[26] Wait, L. F., Dobson, A. P. & Graham, A. L. Do parasite infections interfere with immunisation? A review and meta-analysis. *Vaccine* **38**, 5582–5590. 10.1016/j.vaccine.2020.06.064 (2020). 32616328 10.1016/j.vaccine.2020.06.064

[27] van Dorst, M. M. A. R. et al. Immunological factors linked to geographical variation in vaccine responses. *Nat. Rev. Immunol.* **24**, 250–263. 10.1038/s41577-023-00941-2 (2024). 37770632 10.1038/s41577-023-00941-2

[28] Cooper, P. J. et al. Human infection with *Ascaris lumbricoides* is associated with suppression of the interleukin-2 response to recombinant cholera toxin B subunit following vaccination with the live oral cholera vaccine CVD 103-HgR. *Infect. Immun.* **69**, 1574–1580. 10.1128/iai.69.3.1574-1580.2001 (2001). 11179329 10.1128/IAI.69.3.1574-1580.2001 PMC98058

