# Supplementary material for: Association between helminth infection and impaired SARS-CoV-2 antibody responses following COVID-19 vaccination: a cross-sectional study in Malawi
Source: Sci Rep. 2026 Jul 24;16:26324. doi: 10.1038/s41598-026-62359-9 (PMC13498588; doi:10.1038/s41598-026-62359-9)
Supplement: Supplementary file 1 [file 41598_2026_62359_MOESM1_ESM.pdf]

Supplementary Materials

Association between helminth infection and impaired SARS-CoV-2 antibody responses following COVID-19 vaccination: a cross-sectional study in Malawi.

Authors:

*Mhairi J. McCormack BSc (Hons)[1], m.mccormack.1@research.gla.ac.uk
Louis Banda MSc[2], louis.banda@meiru.mw
Stephen Kasenda MBBS[2], stephen.kasenda@meiru.mw
Lyson Samikwa BSc (Hons)[3], lsamikwa@kuhes.ac.mw
Harry Meleke BSc (Hons)[3], hmeleke@kuhes.ac.mw
Ellen C. Hughes PhD[1,4], ellen.hughes@ed.ac.uk
Amelia Crampin MSc[2,5], mia.crampin@lshtm.ac.uk
David Chaima PhD[3], dchaima@kuhes.ac.mw
Tonney Nyirenda PhD[3], tnyirenda@kuhes.ac.mw
Brian J Willett PhD[1†], Brain.Willett@glasgow.ac.uk
Abena S. Amoah PhD[2,6,7†], a.s.amoah@lumc.nl
Antonia Ho PhD[1†], Antonia.Ho@glasgow.ac.uk
*Corresponding author
[†]These authors contributed equally to this work

Affiliations:
1. Medical Research Council-University of Glasgow Centre for Virus Research, Glasgow, UK
2. Malawi Epidemiology and Intervention Research Unit (MEIRU), Malawi
3. Kamuzu University of Health Sciences (KUHeS), Blantyre, Malawi
4. The Roslin Institute and R(D)SVS, University of Edinburgh, Edinburgh, UK
5. School of Health and Wellbeing, University of Glasgow, Glasgow, UK
6. Department of Population Health, Faculty of Epidemiology and Population Health, London School of Hygiene and Tropical Medicine
7. Leiden University Center for Infectious Diseases, Leiden University Medical Center, Leiden, Netherlands

Table of Contents

Supplementary Methods **3**

Baseline demographic details........ 3

Cells 4

Generation of HIV(SARS-CoV-2) pseudotypes........ 4

Pseudotyped virus neutralisation assay protocol 4

Nucleocapsid ELISA protocol 5

Spike ELISA protocol 5

Helminth detection protocol 6

Statistical analyses 6

Generalised additive model (GAM) 7

Supplementary Tables **10**

Supplementary Table 1. Spike gene construct mutations, relative to the Wuhan-Hu-1 sequence (GenBank: MN908947). 10

Supplementary Table 2. Primers and probes for Schistosoma spp. helminth PCR targeting the internal transcribed spacer (ITS) region. 10

Supplementary Table 3. Participant Characteristics. 11

Supplementary Table 4. Cross tabulation of Schistosoma spp. infection with prevalence of contact with water. 12

Supplementary Table 5. Relationship between nAb titre, helminth status, and vaccination status – all SARS-CoV-2 nAb positives. 12

Supplementary Table 6. Relationship between nAb titre and age (by vaccine status) – all SARS-CoV-2 nAb positives. 13

Supplementary Table 7. Relationship of nAb titre with titre variant and helminth status – vaccinated individuals. 13

Supplementary Table 8. Relationship between nAb titre vs age and titre vs time since COVID-19 vaccination– vaccinated individuals. 14

Supplementary Figures **15**

Supplementary Figure 1. Map of Malawi, showing the location of the Karonga Health Demographic Surveillance Site and Area 25, Lilongwe. 15

Supplementary Figure 2. Helminth prevalence (%) by age group (years) of the participants. Helminth prevalence includes individuals tested positive for 'any helminth infection.' 16

Supplementary Figure 3. Helminth prevalence (%) by biological sex of the participants. Helminth prevalence includes individuals tested positive for 'any helminth infection.' 17

Supplementary Figure 4: Neutralisation of different SARS-CoV-2 viruses in helminth-infected individuals, stratified by helminth type - soil transmitted helminth (STH) (A. duodenale and N. americanus) vs Schistosoma spp. (in stool and urine). 18

Supplementary Figure 5: Relationship between age (years) and nAb titre - all SARS-CoV-2 nAb positives. 19

Supplementary Figure 6: Relationship between nAb titre and time since last vaccination (days) – vaccinated individuals. 20

Supplementary Figure 7: Anti-SARS-CoV-2 Spike IgG (Absorbance) by helminth status, among those Spike (S) ELISA positive, separated by SARS-CoV-2 exposure history. 21

References **22**

Supplementary Methods

Baseline demographic details

Baseline demographic details collected included: socioeconomic indicators; medical history; COVID-19 symptoms; COVID-19 vaccination history; and infection preventative behaviours. Nutritional status information (BMI, waist, hip and arm circumference) was not analysed as it was only collected for 178 (73.6%) of the participants from Karonga, and was not collected for participants from Lilongwe. A questionnaire on helminth treatment, potential helminth infection symptoms, and water contact history was completed.

Sample size

The sample size was primarily limited by funding, which restricted recruitment to approximately 400 participants. A total of 411 individuals were initially included in the metadata. Participants who self-reported living with HIV were excluded due to potential interference with HIV-based retrovirus PVNA systems, as were those whose serum samples were not received in Glasgow, resulting in 378 eligible participants. Of these, six individuals were not tested for helminth infection, and one had insufficient serum for neutralisation assays, leaving 371 participants with complete datasets.

Among the 371 participants, 208 (56.1%) had detectable SARS-CoV-2 nAb titres, of whom 203 had sufficient remaining serum for subsequent titration analyses. Within this subset, 23 participants were helminth-infected and 180 were helminth-uninfected, forming the primary comparison groups for nAb titres (the primary variable of interest).

Given these group sizes, the study was powered only to detect moderate to large differences in SARS-CoV-2 nAb titres. Based on a two-sample t-test framework ($\alpha=0.05$, 80% power), the minimum detectable standardised effect size (Cohen's d) was approximately 0.62. Cohen's d expresses the difference between group means in units of the pooled standard deviation; in this study, the pooled SD of nAb titres was 2,555, corresponding to a detectable absolute difference of approximately 1,584 units. Using conventional Cohen's d benchmarks (0.2 = small, 0.5 = moderate, 0.8 = large), this effect size falls between moderate and large. As nAb titres varied by viral variant, the corresponding detectable differences also differed, ranging from approximately 816 units for Omicron BA.2 to 2,232 units for the ancestral B.1 virus. While smaller differences may still exist, the study was not well powered to detect them. Variant-specific subgroup analyses were performed, though the study was primarily powered for the overall comparison between helminth-infected and helminth-uninfected participants.

Cells

Cells were maintained at 37°C, 5% CO_2. HEK293-ACE2 cells (produced by stable transfection of HEK293 cells with pSCRPSY-hACE2) were kindly provided by Matt Turnbull and Suzannah Rihn, MRC-University of Glasgow Centre for Virus Research. These cells were maintained in Dulbecco's modified Eagle's medium (DMEM) with 10% foetal bovine serum, 2 mM L-glutamine, 100 µg/ml streptomycin, and 100 IU/ml penicillin added (complete DMEM), supplemented with 2 µg/ml puromycin. HEK293T cells (produced by stable transfection of HEK293 cells with a plasmid encoding the simian vacuolating virus 40 (SV40) large T antigen) were obtained from the American Type Culture Collection (ATCC) via University College London. These cells were maintained in complete DMEM supplemented with 400 µg/ml G418.

Generation of HIV(SARS-CoV-2) pseudotypes

To generate HIV-based SARS-CoV-2 pseudotypes (HIV(SARS-CoV-2)), HEK293T cells were transfected with three different plasmids using polyethylenimine (PEI; 1 µL/mL, Polysciences, Warrington, USA): 0.15 µg/mL of a codon-optimized SARS-CoV-2 spike gene expression vector representing one of six variants—Ancestral B.1 (D614G), Alpha (B.1.1.7), Beta (B.1.351), Delta (B.1.617.2), Omicron BA.1 (B.1.1.529), or Omicron BA.2—synthesized by GenScript Biotech (Supplementary Table 1); 0.1 µg/mL of p8.91, a plasmid encoding HIV-1 gag-pol (Addgene) [1]; 0.1 µg/mL of pCSFLW, encoding firefly luciferase [2]. The DNA:PEI complexes were incubated at room temperature for 20 minutes before being added to HEK293T cells. After 48 hours of incubation at 37 °C, culture supernatants containing HIV(SARS-CoV-2) pseudotypes were collected, filtered through 0.45 µm filters, aliquoted, and stored at −80 °C.

Pseudotyped virus neutralisation assay protocol

All serum samples were tested in a single dilution screen for neutralising activity against the Ancestral B.1, Beta, Delta, and Omicron BA.1 spikes (Supplementary Table 1). Sera were diluted 1 in 25 in complete DMEM and incubated in duplicate for one hour with a specific pseudovirus at 37 °C (final serum dilution 1 in 50). HEK293-ACE2 target cells were added and after incubation for 48-72 hours at 37°C, luciferase activity was measured by adding 75µL of Steadylite Plus chemiluminescence substrate to each well (diluted 1 in 3 in distilled water) (Revvity, Waltham, MA, USA) and luminescence was measured using a Revvity EnSight multimode plate reader (Revvity, Waltham, MA, USA). Samples with percent neutralisation >90% were considered positive. Each plate included a no serum control (complete DMEM), a negative control (from a single pre-pandemic serum sample, Scottish National Blood Transfusion Service protocol NATF 765 10), and a positive control (pooled SARS-CoV-2–positive sera from PCR positive individuals, NHS Greater Glasgow and Clyde Biorepository application 550).

Positive samples were titrated against Ancestral B.1, Beta, Delta, Omicron BA.1 and Omicron BA.2 (the variants thought to have been transmitted in Malawi prior to sample collection [3]). Titration was performed by serial dilution of the samples in complete DMEM before a one hour incubation in triplicate with the pseudovirus at 37°C. HEK293-ACE2 target cells

were added and after 48-72 hours, Steadylite Plus chemiluminescence substrate was added and luciferase activity was measured on the EnSight multimode plate reader. Titres were calculated at 50% reduction in infectivity, allowing observation of weaker nAb responses.

Nucleocapsid ELISA protocol

IgG responses to the SARS-CoV-2 nucleocapsid protein were measured using a SARS-CoV-2 N ELISA carried out at the MRC-University of Glasgow Centre for Virus Research, following previously described methods [4].

Immulon 2HB 96-well plates (ThermoFisher Scientific, UK) were coated overnight at 4 °C with 50 µL per well of nucleocapsid (N) protein (1.0 µg/mL in phosphate-buffered saline [PBS]). Plates were washed using a Wellwash Versa plate washer (ThermoFisher Scientific, UK) with PBS containing 0.05% Tween-20 (PBST), then blocked for 1 hour at room temperature with PBST containing 10% casein (Vector Laboratories via 2BScientific, UK). Test and control sera were diluted 1 in 100 in blocking buffer and added in duplicate (50 µL per well), followed by a 1-hour incubation. After washing, 50 µL of horseradish peroxidase (HRP)-conjugated anti-human IgG (Bethyl Laboratories via Cambridge Bioscience, UK) diluted 1 in 3000 was added to each well and incubated for 1 hour. Plates were washed again before addition of 50 µL of 3,3′,5,5′-tetramethylbenzidine (TMB) substrate (KPL, USA) for 10 minutes in the dark. The reaction was stopped with 50 µL of 1 M sulfuric acid, and absorbance was measured at 450 nm using a Multiskan FC plate reader (ThermoFisher Scientific, UK). Raw absorbance values were normalised using the following equation:

$$\text{Normalised absorbance} = \frac{\text{sample absorbance} - \text{negative control mean}}{\text{negative control mean}}$$

Samples with a normalised absorbance >=3·666 were considered positive. The cutoff was selected using receiver operating characteristic (ROC) analysis undertaken with the normalised absorbance values of positive and negative control samples from Malawi (n=200) tested on the assay. For sample testing, each 96-well plate included a no serum control, a negative control, and a positive control (as above for the pseudotyped virus neutralisation assay).

Spike ELISA protocol

IgG responses to the SARS-CoV-2 spike protein were measured using a SARS-CoV-2 S1 ELISA carried out by the MEIRU laboratory, following previously described methods [3,5]. In brief, Immulon 2HB 96-well plates (ThermoFisher Scientific, UK) were coated with 50 µL of recombinant SARS-CoV-2 S1 antigen (www.nibsc.org) at 1 µg/mL in PBS. The assay procedure followed the same steps as the N ELISA. Absorbance was read immediately at 450 nm using an Accuris SmartReader 96 microplate reader (Benchmark Scientific, Edison, NJ, USA), and samples with a normalised absorbance of ≥1.620 were considered positive. Each plate included a single positive serum control and five negative serum controls obtained from MEIRU FIND study samples collected in January 2019 (NHSRC protocol #16/09/1668).

Helminth detection protocol

For urine samples:
Urine samples were tested for *Schistosoma* spp. infection. Genomic DNA was extracted from 200 µL of urine using the FastDNA™ Spin Kit for Soil (MP Biomedicals, Santa Ana, CA), following the manufacturer's instructions. Mechanical lysis was performed using a PowerLyzer 24 high-speed homogenizer (Qiagen, Hilden, Germany). Real-time PCR targeted a 77-base pair fragment of the internal transcribed spacer 2 (ITS2) region, amplified using *Schistosoma*-specific primers Ssp48F (Sch_ITS_48_Fwd) and Ssp124R (Sch_ITS_124_Rev), with detection via the dual-labelled probe Ssp78T (Sch_Probe) (Supplementary Table 2). This method was previously published and validated for use in stool and urine samples [6-8]. Each 7 µL reaction consisted of 2 µL DNA template, 3.5 µL TaqPath ProAmp Master Mix (ThermoFisher Scientific, Waltham, MA), with primers and probe added at final concentrations of 0.25 µM and 0.12 µM, respectively. Thermal cycling was carried out on a QuantStudio 7 Flex Real-Time PCR System (Applied Biosystems, Carlsbad, CA) with the following conditions: 50°C for 2 minutes, 95°C for 10 minutes, followed by 40 cycles of 95°C for 15 seconds and 59°C for 1 minute. A microscopy-confirmed *Schistosoma haematobium (S. haematobium)*-positive urine sample served as the positive control and was processed alongside test samples during extraction and included in each PCR run. Note that *Schistosoma* spp. infections detected in urine are most likely due to *S. haematobium*. However, the PCR assay used detects *Schistosoma* at the genus level and is not species-specific.

For stool samples:
Stool samples were tested for infections by *Schistosoma* spp. and the following STHs: *Ascaris lumbricoides* (*A. lumbricoides*, roundworm), *Ancylostoma duodenale* (*A. duodenale*, hookworm), *Necator americanus* (*N. americanus*, hookworm), and *Trichuris trichiura* (*T. trichiura*, whipworm). Genomic DNA was extracted from approximately 0.25 g of stool using the FastDNA™ Spin Kit for Soil (MP Biomedicals, Santa Ana, CA) and a high-speed homogenizer (PowerLyzer 24, Qiagen, Hilden, Germany), following the manufacturer's protocol. PCR amplification was conducted as described above for urine samples, utilising the same *Schistosoma*-specific primers and probes (Supplementary Table 2) [6-8]. Note that *Schistosoma* spp. infections detected in stool are most likely due to *Schistosoma mansoni (S. mansoni)*. Again, the PCR assay used detects *Schistosoma* at the genus level and is not species-specific. Primer and probe sets for the STHs (specifically *A. lumbricoides, A. duodenale, N. americanus,* and *T. trichiura)* were adopted from Pilotte, et al. [9].

A cycle threshold (Ct) cutoff of 40 was used to define qPCR positivity.

Statistical analyses

Descriptive statistics were calculated to characterise the study participants. Continuous variables were summarised using medians and interquartile ranges (IQRs), as the distributions deviated from normality. The distribution of these variables was assessed visually through histograms and boxplots. Comparisons of continuous variables between independent groups were conducted using the Wilcoxon (rank-sum) test, a non-parametric test appropriate for

data that are not normally distributed. Categorical variables were presented as counts and corresponding percentages. Group comparisons for categorical variables were performed using either the Pearson chi-squared test or Fisher's exact test, depending on the expected frequencies within contingency tables. Specifically, the chi-squared test was employed when all expected cell counts were ≥5, while Fisher's exact test was used when any expected cell count was <5, to ensure statistical validity.

To estimate the effect size of overall helminth-infected/uninfected comparisons, Cliff's Delta was calculated. To assess the power of the Wilcoxon test for detecting differences in titres between groups, a bootstrap simulation approach was used. Specifically, 1,000 bootstrap samples were generated by resampling with replacement within each helminth status group, preserving group sizes. For each bootstrap sample, the Wilcoxon rank-sum test was performed, and the proportion of tests with a p-value < 0.05 was calculated to estimate the statistical power.

A linear model was used to visualise the comparison between helminth PCR Ct values and SARS-CoV-2 nAb titres, with the relationship quantified using Spearman's correlation coefficient (as titres are not normally distributed).

Generalised additive model (GAM)

The relationship between SARS-CoV-2 nAb responses and several predictor variables were assessed. A GAM was chosen following initial analysis using a linear regression model displayed poor predictive power and indicated the presence of non-linear relationships. In the GAM (performed using the package mgcv version 1.9-1 [10] in RStudio), titres were $\log_3$(Titre/50) transformed as the serial dilution for the titrations were performed in 3-fold steps, starting at a 1 in 50 dilution. We generated two models, the first looking at all SARS-CoV-2 nAb positives (vaccinated and infected). The second looked at those vaccinated to allow the inclusion of the variables "time since last vaccination" and "vaccine type".

The GAM including all SARS-CoV-2 nAb positives (vaccinated and infected) examined the influence of following variables that we hypothesise may influence SARS-CoV-2 neutralisation. We ensured that the variables did not correlate with each other using Spearman's rank correlation coefficient to avoid collinearity in the model.

- Biological sex - categorical
- Number of comorbidities - categorical
- Age - continuous
- Helminth status and COVID-19 vaccination status – categorical, combined
- Variant titred against - categorical

The combined term (helminth status and COVID-19 vaccination status) was included to assess the effect of these terms simultaneously, using the interaction symbol ':' when generating the model.

Before fitting the model, Spearman’s rank correlation coefficient was used to check for collinearity among predictor variables, ensuring that highly correlated variables were not included together, which could undermine the model’s validity. An initial model including all candidate predictors was then fitted. Model selection was conducted using backward elimination, removing terms that were not statistically significant in a stepwise manner. This approach helped simplify the model and reduce the risk of overfitting, which was particularly important given the relatively small sample size. Due to sample size constraints, the final optimized model was limited to the two most significant and relevant predictors, ensuring sufficient counts within each subgroup [11].

Model assumptions and fit were assessed using standard GAM diagnostic plots. Quantile–quantile (QQ) plots of deviance residuals were examined to determine whether the residuals followed the expected error distribution. Residuals were also plotted against the linear predictor, and histograms of residuals were inspected to identify any systematic patterns or violations of model assumptions, such as non-linearity, heteroscedasticity, or outliers. Additionally, observed versus fitted values plots were used to evaluate how well the model predictions matched the actual data. The effective degrees of freedom for each smooth term particularly for age, were checked to prevent overfitting, and visual inspection of the smooth functions ensured that the modelled relationships were biologically reasonable.

For the GAM including those COVID-19 vaccinated (either "vaccinated only" or "vaccinated and infected"), the following variables were considered:

- Biological sex - categorical
- Number of comorbidities - categorical
- Age - continuous
- Variant titred against - categorical
- Helminth status - categorical
- Vaccine type - categorical
- Time since last vaccination - continuous

For this, smoothing functions were present on the variables age and time since last vaccination. As above, the non-significant variables were removed and only the two most significant variables were chosen to avoid model overfitting.

Categorical predictor variables were modelled as fixed effects, with coefficients estimated relative to a reference category. The output included coefficient estimates – using the log3(titre/50) scale, standard errors, t-values, and p-values. For ease of interpretation, 95% confidence intervals for the coefficient estimates were calculated using:

$$\text{Lower bound} = \text{Coefficient estimate} - 1.96 \times SE$$
$$\text{Upper bound} = \text{Coefficient estimate} + 1.96 \times SE$$

Additionally, the percentage change in nAb titre was calculated using:

$$\% \text{ change} = (3^{\text{Coefficient estimate}} - 1) \times 100,$$

with the 95% CI calculated as follows:

$$\text{Lower } \% \text{ change} = (3^{\text{Coefficient estimate} - 1.96 \times \text{SE}} - 1) \times 100$$
$$\text{Upper } \% \text{ change} = (3^{\text{Coefficient estimate} + 1.96 \times \text{SE}} - 1) \times 100$$

These values indicate the direction and magnitude of the effect for each category compared to the reference.

Continuous predictor variables were modelled using splines to fit non-linear, smooth relationships between the predictor and nAb titres. The output of this was the estimated degrees of freedom, degrees of freedom, F-statistic and p-value (F-test). For ease of interpretation, chi-squared (χ^2) was calculated using:

$$\chi^2 = F \times \text{estimated degrees of freedom}$$

to give a measure of how strongly the smooth term contributed to the model. If χ^2 was notably higher than the estimated degrees of freedom, it generally indicated a stronger effect of that smooth term in the model. Additionally, the smooth term was plotted.

Categorical predictor variables (factorial) were modelled as fixed effects. The output for these terms consisted of the coefficient estimate on the $\log_3$(titre/50) scale (relative to a reference group), standard error (SE), T-statistic and p-value (Wald test).

Supplementary Tables

Supplementary Table 1. Spike gene construct mutations, relative to the Wuhan-Hu-1 sequence (GenBank: MN908947).

SARS-CoV-2 virus	Mutations relative to Wuhan-Hu-1 sequence
Ancestral (B.1)	D614G
Beta (B.1.351)	D80A, D215G, L241del, L242del, A243del, K417N, E484K, N501Y, D614G, A701V
Delta (B.1.617.2)	T19R, G142D, Δ156-157, R158G, L452R, T478K, D614G, P681R, D950N
Omicron BA.1 (B.1.1.529)	A67V, Δ69–70, T95I, G142D/Δ143–145, Δ211/L212I, ins214EPE, G339D, S371L, S373P, S375F, K417N, N440K, G446S, S477N, T478K, E484A, Q493R, G496S, Q498R, N501Y, Y505H, T547K, D614G, H655Y, N679K, P681H, N764K, D796Y, N856K, Q954H, N969K, L981F
Omicron BA.2	T19I, ∆24/26, G142D, V213G, G339D,S371F, S373P, S375F, T376A, D405N, R408S, K417N, N440K, S477N, T478K, E484A, Q493R, Q498R, N501Y, Y505H,D614G, H655Y, N679K, P681H, N764K, D796Y, Q954H, N969K

Supplementary Table 2. Primers and probes for *Schistosoma* spp. helminth PCR targeting the internal transcribed spacer (ITS) region.
Primers and probe were based on conserved ITS sequences across *Schistosoma* species.

	Primer name	Sequence 5'-3'
**Schistosoma spp.**	Sch_ITS_48_Fwd	GGTCTAGATGACTTGATYGAGATGCT
	Sch_ITS_124_Rev	TCCCGAGCGYGTATAATGTCATTA
	Sch_Probe	FAM-TGGGTTGTGCTCGAGTCGTGGC-BHQ1

Supplementary Table 3. Participant Characteristics.

	Karonga (rural) (n=242)	Lilongwe (urban) (n=129)	P-value
**Median age (IQR), years**	36.1 (16.0-50.0)	38.2 (28.3-49.2)	0.49
..	..	..	..
**Age category, years - n (%)**	..	..	0.97
18-39	139 (57.4)	73 (56.6)	..
40-59	75 (31.0)	41 (31.8)	..
≥60	28 (11.6)	15 (11.6)	..
..	..	..	..
**Sex - n (%)**	..	..	0.002
Female	129 (53.3)	91 (70.5)	..
Male	113 (46.7)	38 (29.5)	..
..	..	..	..
**Received >=1 COVID-19 vaccinations (n, %)**	77 (31.8)	45 (34.9)	0.63
..	..	..	..
**Treatment for schistosomiasis (n, %)**	..	..	<0.0001
Never	131 (54.1)	96 (74.4)	..
Once	37 (15.3)	5 (3.9)	..
Twice	17 (7.0)	2 (1.6)	..
>=3 times	52 (21.5)	18 (13.9)	..
Unknown	5 (2.1)	8 (6.2)	..
..	..	..	..
**Number reporting symptoms in past 2 weeks (n, %)**	..	..	..
Blood in urine	18 (7.4)	10 (7.8)	1.00
Pain when passing urine	34 (14.0)	12 (9.3)	0.25
Blood in stool	14 (5.8)	9 (7.0)	0.82

IQR = interquartile range, COVID-19 = coronavirus disease 2019.

Supplementary Table 4. Cross tabulation of *Schistosoma* spp. infection with prevalence of contact with water.

	Daily	4-6 times per week	1-3 times per week	Once a month	Once a year	No contact with a water body
***Schistosoma* spp. uninfected**	151 (44.3%)	8 (2.3%)	37 (10.9%)	5 (1.5%)	1 (0.3%)	139 (40.8%)
..	..	..	..	..	..	..
***Schistosoma* spp. infected**	13 (43.3%)	1 (3.3%)	4 (13.3%)	0 (0.0%)	1 (3.3%)	11 (36.7%)

Shown as n participants with % per *Schistosoma* spp. status.

Supplementary Table 5. Relationship between nAb titre, helminth status, and vaccination status – all SARS-CoV-2 nAb positives. A GAM was used to model the relationships between predictors and nAb titres. Coefficient estimate shows the extent to which the variable influences nAb titre (on the $\log_3(x/50)$ scale) with a positive value for an increase, negative value for a decrease. 95% confidence interval (CI) shows the variability in this measure. Percent (%) change represents the change in titre associated with the specific condition. P-value shows the statistical significance and interpretation summarises the output. 203 participants included. Baseline reference categories – COVID-19 unvaccinated, helminth-uninfected.

Variable	Coefficient estimate (95% CI)	% change (95% CI)	P-value	Interpretation
**Intercept**	0.63 (0.57 to 0.68)	NA	NA	Baseline level of the outcome (nAb titre) when all predictors are at their reference category – COVID-19 unvaccinated and helminth-uninfected.
**Helminth status: infected:COVID-19 vaccination:vaccinated**	-0.53 (-0.80 to -0.25)	-41% (-55% to -22%)	0.00024	Individuals vaccinated and helminth-infected have lower titres than non-infected vaccinated individuals

CI = confidence interval; COVID-19 = coronavirus disease 2019; nAb = neutralising antibody; NA = not applicable.

Supplementary Table 6. Relationship between nAb titre and age (by vaccine status) – all SARS-CoV-2 nAb positives. Estimated degrees of freedom indicates the flexibility of the smooth function (1 is a linear relationship, higher values represent increasing non-linearity). Reference degrees of freedom are the number of degrees of freedom set for testing. χ^2 tests whether the smooth term explains variation in neutralisation levels (higher values for stronger effects). P-value shows the statistical significance and interpretation summarises the output (F-test). 203 participants included. Note for this model a k of 6 was used to avoid overfitting.

Smooth term	Estimated degrees of freedom	Reference degrees of freedom	χ^2	P-value	Interpretation
**Age**	1	1.001	30.3	<0.0001	Linear relationship between age and nAb titre among those unvaccinated

χ^2 = chi-squared.

Supplementary Table 7. Relationship of nAb titre with titre variant and helminth status – vaccinated individuals. A GAM was used to model the relationships between predictors and nAb titres. Coefficient estimate shows the extent to which the variable influences nAb titre (on the $\log_3$(Titre/50) scale) with a positive value for an increase or a negative value for a decrease. 95% confidence interval (CI) shows the variability in this measure. Percent (%) change represents the change in titre associated with the specific condition. P-value shows the statistical significance and interpretation summarises the output. Baseline reference category – helminth-uninfected. 94 participants included.

Variable	Coefficient estimate (95% CI)	% change (95% CI)	P-value	Interpretation
**Intercept**	0.72 (0.66 to 0.77)	NA	NA	Baseline level of the outcome (nAb titre) when all predictors are at their reference category
**Helminth status: Infected**	-0.34 (-0.55 to -0.13)	-29% (-41% to -16%)	0.0018	Helminth-infected individuals have lower titres than those helminth-uninfected

CI = confidence interval; nAb = neutralising antibody; NA = not applicable.

Supplementary Table 8. Relationship between nAb titre vs age and titre vs time since COVID-19 vaccination– vaccinated individuals. Estimated degrees of freedom indicates the flexibility of the smooth function (1 is a linear relationship, higher values represent increasing non-linearity). Reference degrees of freedom are the number of degrees of freedom set for testing. χ^2 tests whether the smooth term explains variation in neutralisation levels (higher values for stronger effects). P-value shows the statistical significance and interpretation summarises the output (F-test). 94 participants included

Smooth term	Estimated degrees of freedom	Reference degrees of freedom	χ^2	P-value	Interpretation
**Time since COVID-19 vaccination**	6.72	7.83	46.8	<0.0001	Non-linear relationship between age and nAb titre among those vaccinated

χ^2 = chi-squared; COVID-19 = coronavirus disease 2019.

Supplementary Figures

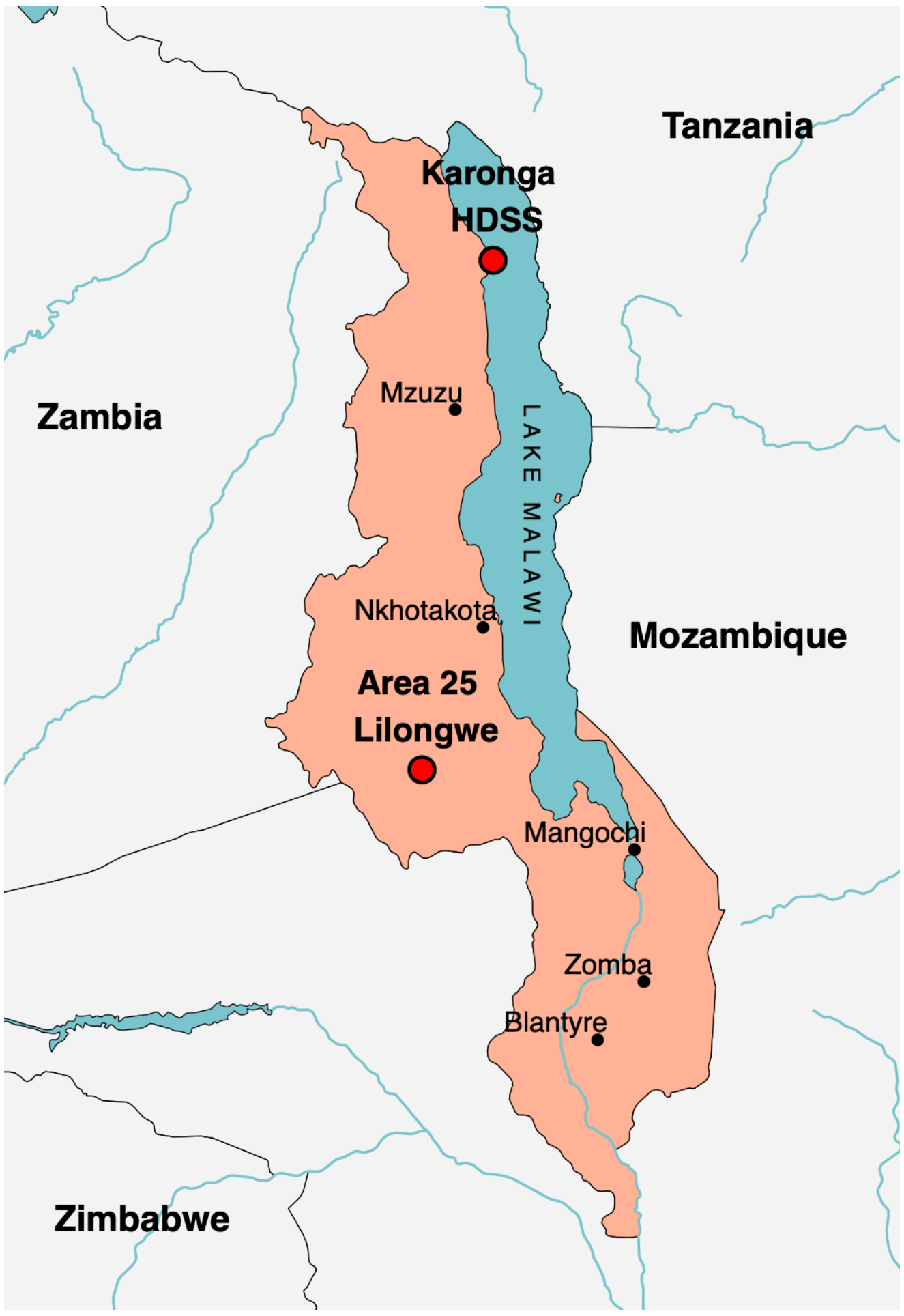

Supplementary Figure 1. Map of Malawi, showing the location of the Karonga Health Demographic Surveillance Site and Area 25, Lilongwe. Red dots are the study locations.

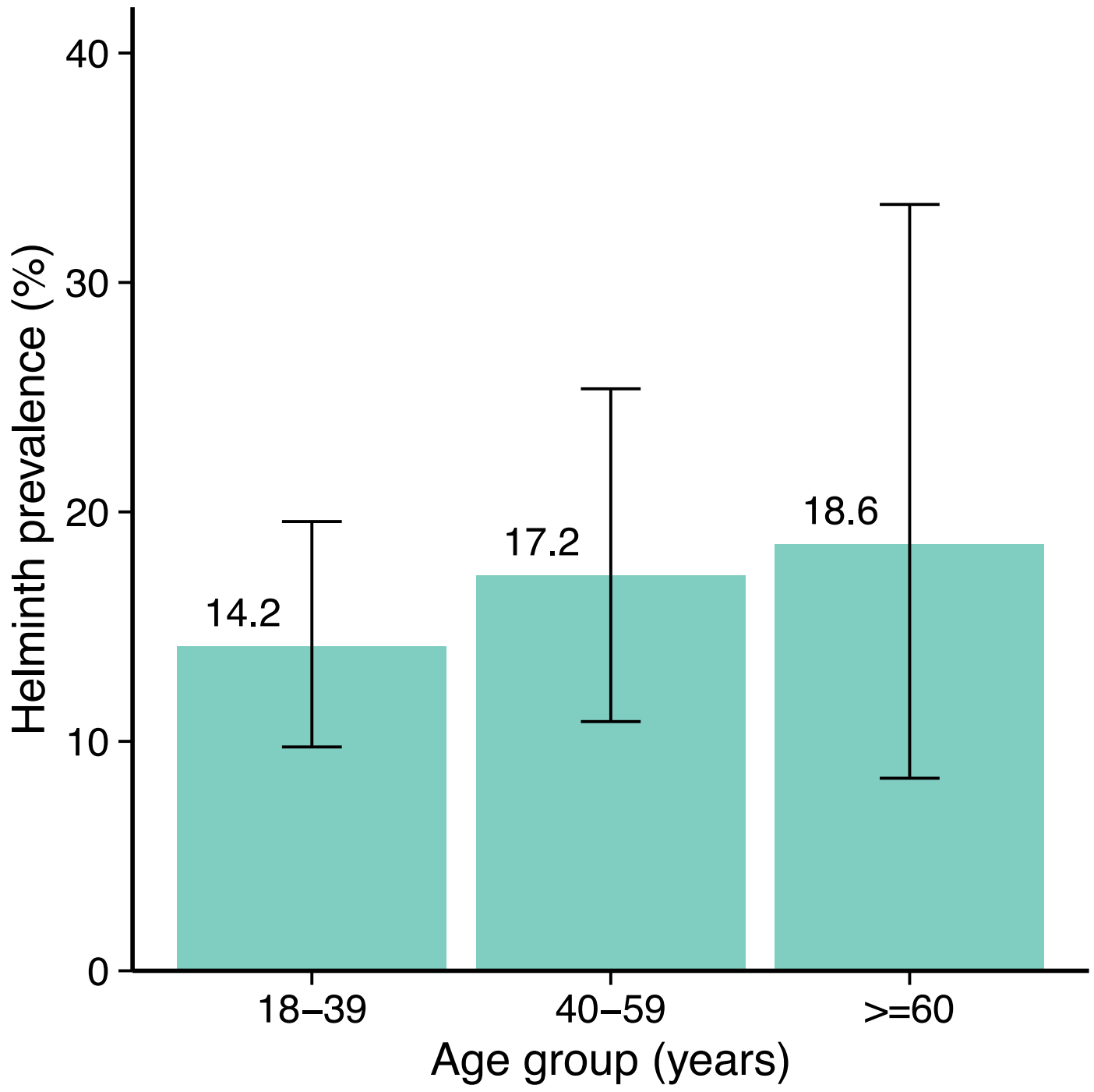

Supplementary Figure 2. Helminth prevalence (%) by age group (years) of the participants. Helminth prevalence includes individuals tested positive for 'any helminth infection.' Bar charts display the percentage of participants in each condition (% labelled as text), with error bars displaying the 95% confidence interval for the prevalence.

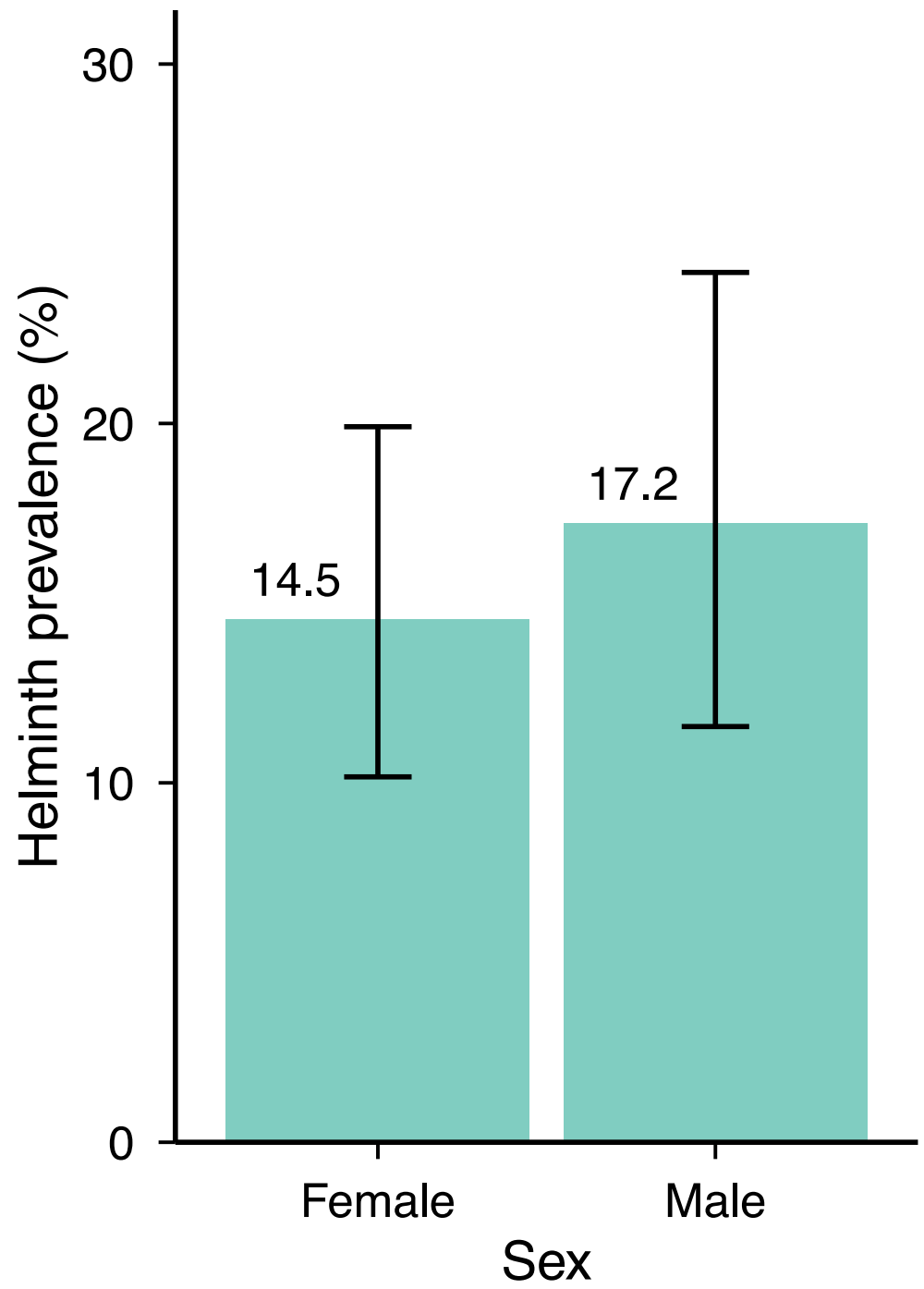

Supplementary Figure 3. Helminth prevalence (%) by biological sex of the participants. Helminth prevalence includes individuals tested positive for 'any helminth infection.' Bar charts display the percentage of participants in each condition (% labelled as text), with error bars displaying the 95% confidence interval for the prevalence.

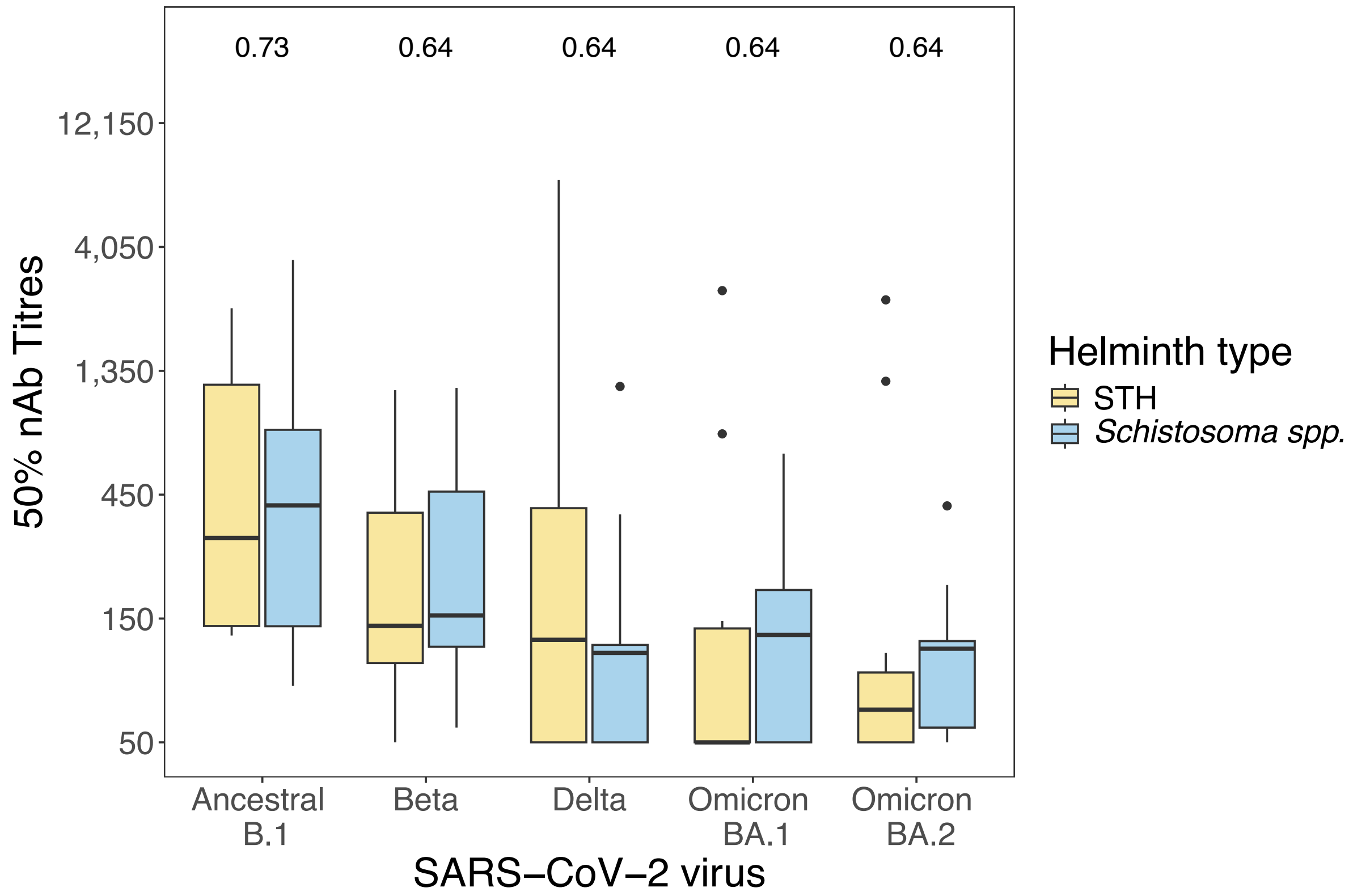

Supplementary Figure 4: Neutralisation of different SARS-CoV-2 viruses in helminth-infected individuals, stratified by helminth type - soil transmitted helminth (STH) (*A. duodenale* and *N. americanus*) vs *Schistosoma* spp. (in stool and urine). 22 participants included – n=13 with STH infection; n=9 with Schistosoma infection. Titres were measured using HIV(SARS-CoV-2) pseudotyped virus neutralisation assay. Box plots display the median and interquartile range (IQR) of the outcome (50% titre). Statistical test used was Wilcoxon rank sum test with a false discovery rate correction applied: p-values are presented.

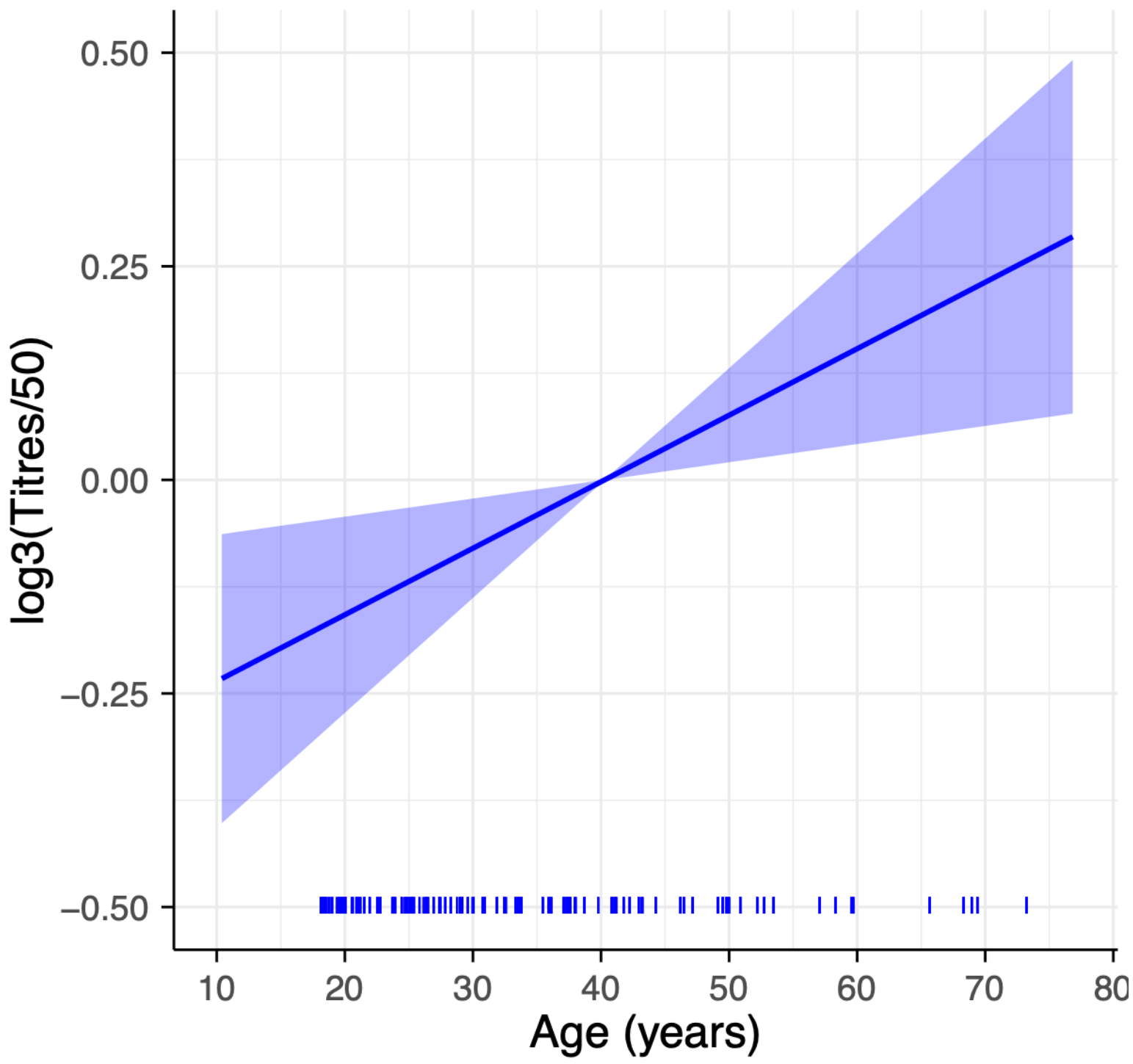

Supplementary Figure 5: Relationship between age (years) and nAb titre - all SARS-CoV-2 nAb positives. The linear relationship between age(years) and nAb titre, determined using a GAM. The solid line represents the estimated smooth function, while the shaded region indicates the 95% confidence intervals for this estimate. The x-axis represents the predictor variable (age) while the y-axis represents the response (neutralising titres - $\log_3$(Titres/50). The rug marks represent the distribution of participants by age in both the unvaccinated and vaccinated groups. 203 participants included.

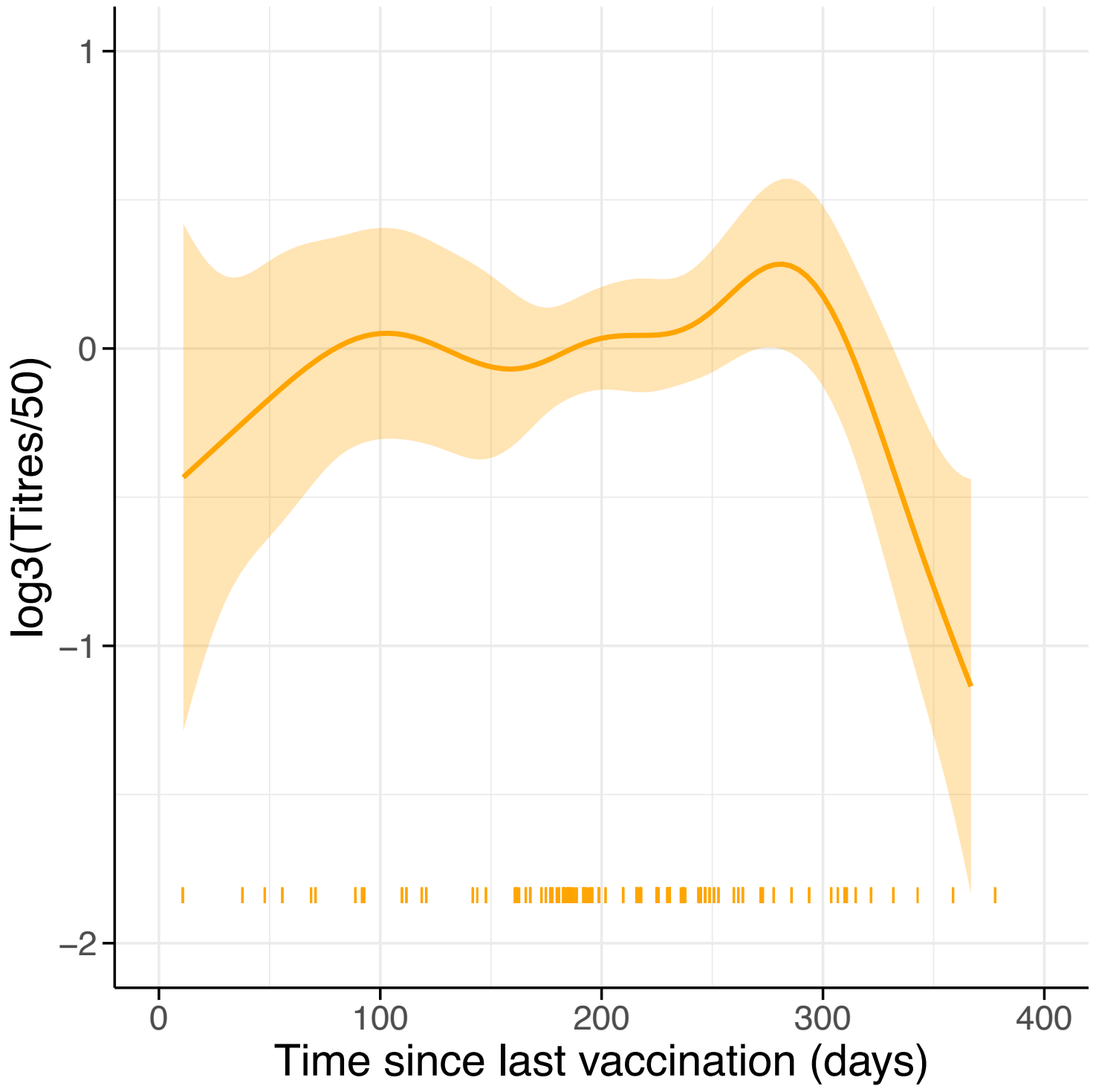

Supplementary Figure 6: Relationship between nAb titre and time since last vaccination (days) – vaccinated individuals. The non-linear relationship between time since last vaccination (days) and nAb titre, determined using a GAM. The solid line represents the estimated smooth function, while the shaded region indicates the 95% confidence intervals for this estimate. The x-axis represents the predictor variable while the y-axis represents the response (neutralising titres - $\log_3$(Titres/50)). The rug marks represent the distribution of participants within the predictor variable. 94 participants included.

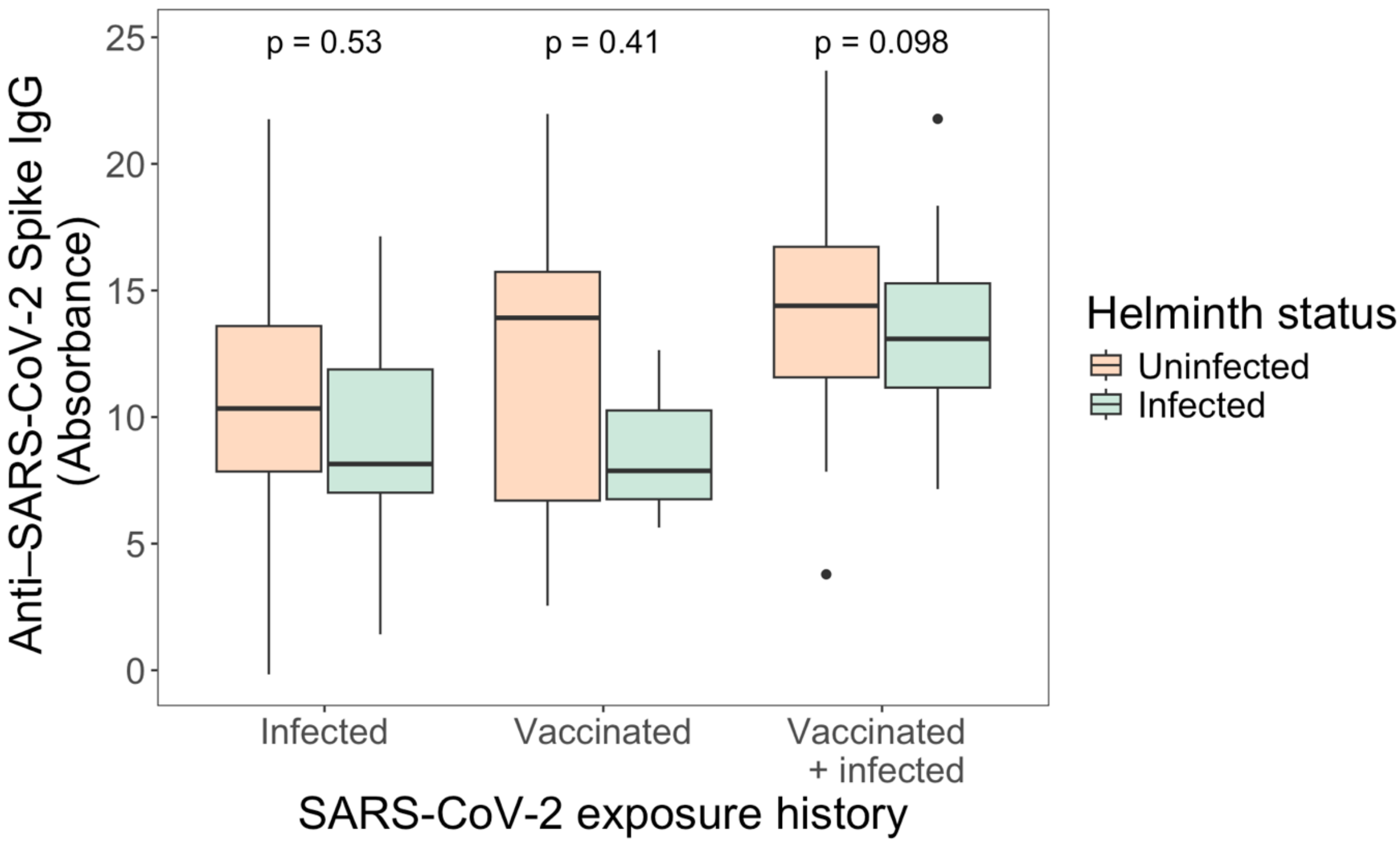

Supplementary Figure 7: Anti-SARS-CoV-2 Spike IgG (Absorbance) by helminth status, among those Spike (S) ELISA positive, separated by SARS-CoV-2 exposure history. Anti-SARS-CoV-2 S IgG measured with an enzyme-linked immunosorbent assay (ELISA) using the ancestral B.1 S protein. Among those SARS-CoV-2 infected, 112 participants were helminth-uninfected and 23 were helminth-infected. Among those COVID-19 vaccinated, 19 participants were helminth-uninfected and 3 were helminth-infected. Among those SARS-CoV-2 infected and COVID-19 vaccinated, 73 participants were helminth-uninfected and 8 were helminth-infected. Boxplots show median and IQR of 50% titres. The whiskers stretch to the farthest data points that lie within 1.5 × IQR of Q1 and Q3, while any points beyond this range are displayed as outliers. Wilcoxon (rank-sum) test with false discovery rate correction was used; p-values shown. Due to very small subgroup sizes, statistical comparisons should be interpreted with caution and are presented as exploratory analyses.

References

1 Zufferey, R., Nagy, D., Mandel, R. J., Naldini, L. & Trono, D. Multiply attenuated lentiviral vector achieves efficient gene delivery in vivo. *Nat. Biotechnol.* **15**, 871-875; 10.1038/nbt0997-871 (1997).

2 Zufferey, R. et al. Self-inactivating lentivirus vector for safe and efficient in vivo gene delivery. *J. Virol.* **72**, 9873-9880; 10.1128/jvi.72.12.9873-9880.1998 (1998).

3 Banda, L. et al. Characterizing the evolving SARS-CoV-2 seroprevalence in urban and rural Malawi between February 2021 and April 2022: A population-based cohort study. *Int. J. Infect. Dis.* **137**, 118-125; 10.1016/j.ijid.2023.10.020 (2023).

4 McCormack, M. J. et al. Characterising protective immune responses to SARS-CoV-2 in urban and rural Malawi between February 2021 and April 2022. *Sci. Rep.* **15**, 37914; 10.1038/s41598-025-22599-7 (2025).

5 Hughes, E. C. et al. Severe acute respiratory syndrome coronavirus 2 serosurveillance in a patient population reveals differences in virus exposure and antibody-mediated immunity according to host demography and healthcare setting. *J. Infect. Dis.* **223**, 971-980; 10.1093/infdis/jiaa788 (2021).

6 Meurs, L. et al. Is PCR the Next Reference Standard for the Diagnosis of Schistosoma in Stool? A Comparison with Microscopy in Senegal and Kenya. PLoS. Negl. Trop. Dis. **9**, e0003959; 10.1371/journal.pntd.0003959 (2015).

7 Aryeetey, Y.A. et al. Molecular Diagnosis of Schistosoma Infections in Urine Samples of School Children in Ghana. Am. J. Trop. Med. Hyg. **88**, 1028-1031; 10.4269/ajtmh.12-0571 (2013).

8 Kenguele, H.M. et al. Impact of Short-Time Urine Freezing on the Sensitivity of an Established Schistosoma Real-Time PCR Assay. Am. J. Trop. Med. Hyg. **90**, 1153-1155; 10.4269/ajtmh.14-0005 (2014).

9 Pilotte, N. et al. Improved PCR-based detection of soil transmitted helminth infections using a next-generation sequencing approach to assay design. *PLOS Negl. Trop. Dis.* **10**, e0004578; 10.1371/journal.pntd.0004578 (2016).

10 Wood, S. N. Fast stable restricted maximum likelihood and marginal likelihood estimation of semiparametric generalized linear models. J. R. Stat. Soc. Ser. A Stat. Soc. **73**, 3-36 (2011).

11 Ogundimu, E. O., Altman, D. G. & Collins, G. S. Adequate sample size for developing prediction models is not simply related to events per variable. *J. Clin. Epidemiol.* **76**, 175-182; 10.1016/j.jclinepi.2016.02.031 (2016).
